# Comparison of Rumen Microbiota and Serum Biochemical Indices in White Cashmere Goats Fed Ensiled or Sun-Dried Mulberry Leaves

**DOI:** 10.3390/microorganisms8070981

**Published:** 2020-06-30

**Authors:** Yaoyue Wang, Qingmiao Shen, Shu Zhong, Yulin Chen, Yuxin Yang

**Affiliations:** 1 College of Animal Science and Technology, Northwest A&F University, Yangling 712100, Shaanxi, China; yaoyue@nwafu.edu.cn (Y.W.); b20193040329@cau.edu.cn (Q.S.); suvi@nwafu.edu.cn (S.Z.); 2 College of Animal Science and Technology, China Agricultural University, Beijing 100083, China

**Keywords:** mulberry leaves, cashmere goats, ruminal microbial communities, serum biochemical indices, 16S rRNA gene sequencing

## Abstract

Mulberry leaves, which have high nutritional value, have not been fully utilized. Few research systems have indicated whether mulberry leaves can replace traditional feed ingredients in goats. In this study, we investigated the effects of feeding white cashmere goats ensiled (Group E) or sun-dried mulberry leaves (Group S) on changes in ruminal microbial communities, rumen fermentation parameters and serum biochemical indices. The control group (Group C) received a typical total mixed ration (TMR). 16S rRNA gene sequencing revealed 209 genera belonging to 19 bacterial phyla dominated by *Firmicutes* and *Bacteroidetes*. Only the relative abundances of *Erysipelotrichaceae*_UCG-009 were significantly different among the three groups (*p* < 0.05). Physiological and biochemical findings revealed that only the serum leptin concentrations were significantly decreased when mulberry leaves were added to the diets (*p* < 0.05). Correlation analysis revealed that *Ruminococcus*_2 were significantly positively correlated with the butyrate concentration. These findings suggested that supplementation with mulberry leaves only induced minor changes in the abovementioned indicators, implying that the rumen fermentation status was still stable after adding mulberry leaves to the diets.

## 1. Introduction

Mulberry has a broad ecological distribution all over the world and shows a strong adaptive ability under drought and cold environments [1]. The area of mulberry cultivation has been reported to be more than 10^6^ ha, and the biomass yield of fresh mulberry leaves has been reported to be 25~30 t/ha/year in China [2]. According to the Tables of Feed Composition and Nutritive in China (2019 Thirty Edition) and published studies from others, mulberry leaves contain a lower neutral detergent fiber (NDF) content (24.6~32.3%), similar crude protein (CP) content (15~28%) and higher ether extract (EE) content (3.5~5.57%) on dry matter (DM) basis compared with alfalfa (34.0~59.0% NDF, 13.0~28% CP and 1.3~3.0% EE), which is one of the most widely cultivated legume forages due to its high CP content [3,4,5,6,7]. The other major feature of mulberry leaves is high palatability, which is related to their moderate concentration of tannins (1.8% as tannic acid equivalent) [8,9]. Studies have reported that the excessive amount of tannins (approximately 3%) in canola seed cake reduced its palatability and nutritional value; however, the insufficient tannins in alfalfa hay induced ruminants’ bloat [10,11]. Additionally, as compared with alfalfa, mulberry leaves have higher DM digestibility in vivo in goats (78.4~80.8% vs. 65.9%) [12,13]. Many studies also have shown that mulberry leaves had hypolipidemic and antioxidant properties due to flavonoids being the most important constituents in leaves [14,15,16]. Although mulberry leaves are rich in nutrients, they are not fully utilized as feed ingredients for ruminants, especially in the diets of goats.

Generally, there are three feed types of mulberry leaves used in livestock: fresh, sun-dried and ensiled. Fresh leaves have a high moisture content but cannot be preserved for a long time. Thus, supplementation with sun-dried or ensiled mulberry leaves is considered in the diets of goats because of the longer storage time of these products. Feed types may influence ruminal microbial communities and the interactions between these communities and their host [17]. However, little is known about the effects of sun-dried and ensiled mulberry leaves on rumen fermentation.

The stability of rumen fermentation does not only affect the rumen health, but also host health [4,18]. Rumen fermentation is an anaerobic process carried out by complex ruminal microbiota, which primarily converts feedstuffs into volatile fatty acids (VFAs), microbial proteins and vitamins [19]. Dietary changes have been shown to be the major determinant of microbial structure and function (fermentation capacity and enzyme activity) in the rumen [20,21]. Previous studies reported that mulberry leaves improved the rumen fermentation in the finishing steers and beef cattle [22,23]. However, few studies have comprehensively explored the effects of mulberry leaves on the rumen microbiota in ruminants, especially in goats.

Serum biochemical indices could reflect that the health status of animal [24] and rumen microbiota, as described above, is important for host health. Thus, accumulated studies have focused on the close relationships between microbiota and serum biochemical indices. Li et al. [25] have shown that the serum low-density lipoprotein cholesterol level had a positive relationship with *Bifidobacterium*. He et al. [26] and Shao et al. [24] have reported that the serum triglyceride levels were negatively associated with the genus *Acinetobacter* and *Rikenellaceae*_RC9_gut_groups, respectively. However, the relationships between rumen microbiota and serum biochemical indices in goats fed mulberry leaves remain elusive.

In this study, 16S rRNA gene sequencing and quantitative real-time PCR (qPCR) were used to assess the effects of mulberry leaves on the rumen microbial composition in white cashmere goats, which were regarded as an important economic resource for local farmers in northwest China (the number of this breed is ≥60 million). To gain a deeper understanding of rumen fermentation in goats, we also elucidated the relationships of the microbial populations with rumen morphological and functional parameters and serum biochemical indices. Thus, this study aimed to comprehensively explore whether the effects of mulberry leaves on rumen fermentation and serum biochemical indices, which were necessary to assess rumen health, resulted in a potential impact on host health. Additionally, this study would lay the foundation for mulberry leaves as a new feed resource in goats.

## 2. Materials and Methods

### 2.1. Ethical Approval

The use of animals and all experimental protocols (protocol number 100403) were authorized by the Institutional Animal Care and Use Committee of Northwest A&F University (Yangling, Shaanxi, China).

### 2.2. Diet, Animal Management, and Sample Collection

All diets were formulated to meet the nutrient requirements for growing goats according to the Feeding Standard for Meat-Producing Sheep and Goats (NY/T816-2004, China) and the results of previous studies from our laboratory. Six dietary treatments were administered (Supplemental Table S1); the control group (Group C) received a typical total mixed ration (TMR); the ensiled mulberry leaf groups (Group E) received a typical TMR supplemented with 10%, 15% or 20% mulberry leaf silage (Groups E1, E2 and E3); and the sun-dried mulberry leaf groups (Group S) received a typical TMR supplemented with 10% or 15% sun-dried mulberry leaves (Groups S1 and S2).

Sixty white cashmere goats (wether, 28 ± 1.05 kg) with an average initial age of 230 days were used in this study. The goats were from the Shuntian Breeding Farm (37.18° N, 109.80° E), located in the northern part of Shaanxi Province in China and were randomly allotted to one of the six dietary treatments (a total of 10 goats in each treatment). The goats had free access to water and were fed twice each day with equal amounts of feed at 8:00 and 18:00 h. The experimental period was 70 days, including the first 10 days for diet adaptation. On the last day, a total of three goats were randomly selected from each treatment, and the eighteen goats were then slaughtered. Rumen tissues (~1 × 1 cm) were then collected from the ventral sac, fixed in 40% paraformaldehyde and stored at 4 °C for morphological determination. The rumen contents (approximately 50 mL/goat) were collected after slaughter, and homogenized with a magnetic stirrer (Dragon Lab MS-H280-Pro, Dragon Laboratory Instruments Limited, Beijing, China) at 200 rpm for 2 min, the pH was immediately measured with a pH meter (testo-206-pH 2, Testo Co., Lenzkirch, Germany). Then, the homogenized samples containing solid and liquid contents, were stored at −80 °C for further analyses. About 10 mL of blood samples per goat were collected from the jugular vein shortly before slaughter and were allowed to clot at room temperature (22~25 °C), then, the samples were centrifuged at 4000 rpm at 4 °C for 5 min to separate serum. All serum samples were frozen at −20 °C for biochemical analyses.

### 2.3. DNA Extraction and 16S rRNA Gene Sequencing

Total genomic DNA extraction was carried out on the rumen samples using a stool DNA kit (OMEGA Bio-Tek, Norcross, GA, USA). Specifically, the V3-V4 hypervariable regions of the bacterial 16S rRNA gene were targeted for amplification using the universal primers V338F and V806R. The reaction mixture (20 µL) (TransGen AP221-02: TransStart FastPfu DNA Polymerase, TransGen Biotech, Beijing, China) consisted of 4 µL of 5 × FastPfu Buffer, 2 µL of 2.5 mM dNTPs, 0.4 µL of FastPfu polymerase, 0.8 µL of each primer and 10 ng of template DNA. Triplicate PCRs were performed for each sample. The reaction conditions consisted of an initial denaturing step for 3 min at 95 °C; followed by 27 cycles of 30 s at 95 °C, 30 s at 55 °C, 45 s at 72 °C; and 10 min at 72 °C. Amplification products were run in 2% agarose gels and purified with the QIAquick Gel Extraction Kit (Qiagen, Hilden, Germany). Purified PCR products were quantified fluorometrically. Equimolar ratios of total products were pooled and sequenced with the Illumina MiSeq platform at Majorbio Bio-Pharm Technology Co., Shanghai, China.

### 2.4. Sequencing Analyses

To normalize the sequencing depth and minimize sequencing artifacts across samples, we randomly selected 29,123 sequencing reads per sample in this study. The sequence reads that passed quality control were analyzed using the QIIME 1.9.1 software package [27]. Sequences were clustered into operational taxonomic units (OTUs) with 97% similarity cut-off using UCLUST [28]. The most abundant sequence within each OTU was defined as the “representative sequence” and then aligned using PyNAST software [29,30]. Taxonomic assignments of the representative sequences were determined using RDP Classifier [27]. Community richness analysis was estimated using the ACE and Chao1 indices, and diversity analysis was performed using the Shannon and Simpson indices. Rarefaction curves were analyzed using MOTHUR [31]. Analysis of similarity (ANOSIM) of Bray–Curtis distances was performed to assess the statistical significance of differences in the bacterial community between different groups of samples. Principal coordinate analysis (PCoA) was also performed to compare the dissimilarities in bacterial community structures in different groups [32]. A Circos graph was generated using Circos software [33]. A heatmap was drawn with the gplots package of R software (version 3.1.2) [34]. Significant interactions between bacterial communities at the genus level were determined using Cytoscape version 3.6.1.

### 2.5. Bacterial Function Prediction

Bacterial functional predictive analysis was carried out via phylogenetic investigation of communities by the reconstruction of unobserved states (PICRUSt) [35]. The predicted genes were clustered and categorized under the Kyoto Encyclopedia of Genes and Genomes database (KEGG) [36]. The raw reads obtained in the current study have been deposited in the NCBI Sequence Read Archive (SRA) database under accession number SRP150333.

### 2.6. qPCR Analysis

Absolute qPCR was performed to enumerate gene copies from anaerobic fungi, methanogens and ciliate protozoa using an iCycle iQ5 thermocycler (Bio-Rad, Hercules, CA, USA). The primers were selected based on the published literature (Supplemental Table S2). External standards were prepared by a 10-fold serial dilution of purified plasmid DNA containing the cloned marker loci. All the obtained standard curves met the required standards of efficiency (R^2^ > 0.99, 120% > E > 90%). The reaction mixture and conditions were as previously described [37], with the exception of the annealing temperature. The annealing temperature in this study were provided in Supplemental Table S2. The total numbers of gene copies were expressed as log_10_ numbers of marker loci gene copies per 10 ng of sample.

### 2.7. Volatile Fatty Acid, NH_3_-N and Carboxymethylcellulase Activity Assays

The VFA concentration was determined by gas chromatography (Agilent 7820A, Agilent Technologies, CA, USA) [38]. The total ammonia–nitrogen (NH_3_-N) concentration was measured as an indicator of protein fermentation using the colorimetric phenol–hypochlorite method [39]. The carboxymethylcellulase (CMCase) activity in the rumen contents was determined by measuring the release of glucose from carboxymethylcellulose, with glucose as the standard. The reaction mixture contained 0.2 mL of enzyme solution and 0.4 mL of 0.5% sodium carboxymethylcellulose (CMC-Na) were incubated at 39 °C for 30 min. After incubation, samples were terminated by the addition of 0.6 mL 3,5-dinitrosalicylic acid reagent. The mixtures were then placed in the boiling water bath for 5 min and cooled down at room temperature. The absorbance of the reaction solutions was measured at 540 nm. One enzyme activity unit (U) was defined as the amount of glucose (μmol) produced by 1 mL of enzyme in one minute (μmol/min·mL) [40].

### 2.8. Rumen Morphological Examination

After fixation in 40% paraformaldehyde, tissue specimens were dehydrated, cleared, embedded in paraffin, cut and stained with hematoxylin and eosin. For each tissue specimen, the papilla length (PL), papilla width (PW), papilla surface area (PSA) and rumen wall thickness (RWT) were examined. Morphometric analyses were performed with Image Pro Plus 6.0 (Media Cybernetics, Silver Spring, MD, USA).

### 2.9. Serum Biochemical Analysis

The serum biomarkers detected in this study are all involved in lipid metabolism. Among them, total cholesterol (TC), low-density lipoprotein cholesterol (LDL-C), high-density lipoprotein cholesterol (HDL-C) and lipoprotein lipase (LPL) are mainly involved in cholesterol metabolism and lipoprotein metabolism [5,41]. Triglycerides (TG), lipase (LPS), leptin and fatty acid synthase (FASN) are mainly related to triglycerides metabolism [42,43]. TC, TG, HDL-C, and LDL-C levels in serum were measured with colorimetry by a BS-420 automatic biochemical analyzer (Shenzhen Mindray Bio-Medical Electronics Co., Ltd., Shenzhen, China). LPS and LPL levels were measured with colorimetry by an A6 semiautomatic biochemical analyzer (Songshang Technology Co., Beijing, China). Serum leptin and FASN concentrations were determined with enzyme immunoassay by a spectrophotometer (Stat Fax-2100, Awareness Technology Inc., Palm City, FL, USA).

### 2.10. Statistical Analysis

Statistical analyses were performed using Statistical Package for the Social Sciences (SPSS) version 20.0 for Windows (SPSS Inc., Chicago, IL, USA). Data were analyzed using one-way analysis of variance (ANOVA). Spearman correlation analysis was employed to assess significant associations among bacterial groups, related fermentation parameters and serum biochemical indices if the correlation coefficients (r, in absolute values) were above 0.55 [19,44]. In all analyses, significance was set at <0.05. Additionally, the corrected-*p* values from the differences in abundances of bacteria at the phylum or genus level, the differences in bacterial function prediction, and the differences in spearman correlation analysis between bacterial genera and biochemical indices were calculated using the fdrtool package in R (version 3.1.2).

## 3. Results

### 3.1. Sequences Across Different Dietary Groups

**Figure 1:**
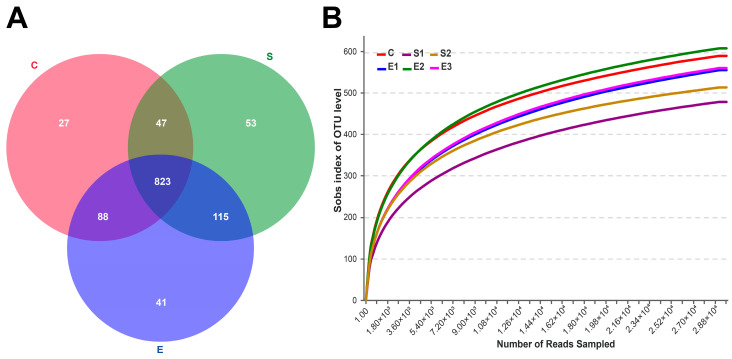
Sequences across different dietary groups (**A**) A Venn diagram illustrating the overlap of bacterial OTUs at a 3% dissimilarity level for Groups C, S and E. Group C samples included goats that were fed a typical total mixed ration (TMR), and Group S samples included goats that were fed a typical TMR supplemented with 10% or 15% sun-dried mulberry leaves (Groups S1 and S2). Group E samples included goats that were fed a typical TMR supplemented with ensiled mulberry leaves (Groups E1, E2 and E3). (**B**) Rarefaction analyses of the different samples. Rarefaction curves of OTUs clustered at 3% divergence.

After quality control, 671,255 valid sequences were obtained from 18 different samples with 29,123 rarefied sequencing reads per sample (Supplemental Table S3). Group S exhibited the largest number of unique sequences (53 OTUs), followed by Group E (41 OTUs) and Group C (27 OTUs). A total of 823 common OTUs (approximately 69% of the total OTUs) were shared among the three groups (Figure 1A).

Good’s coverage (>0.99) and the rarefaction curves at 97% similarity (Figure 1B) indicated that the sampling depth was adequate. Rumen bacterial diversity (Shannon and Simpson indices) and richness (ACE and Chao indices) did not differ under the different dietary treatments (*p* > 0.05) (Supplemental Table S4).

### 3.2. Analysis of Similarity

**Figure 2:**
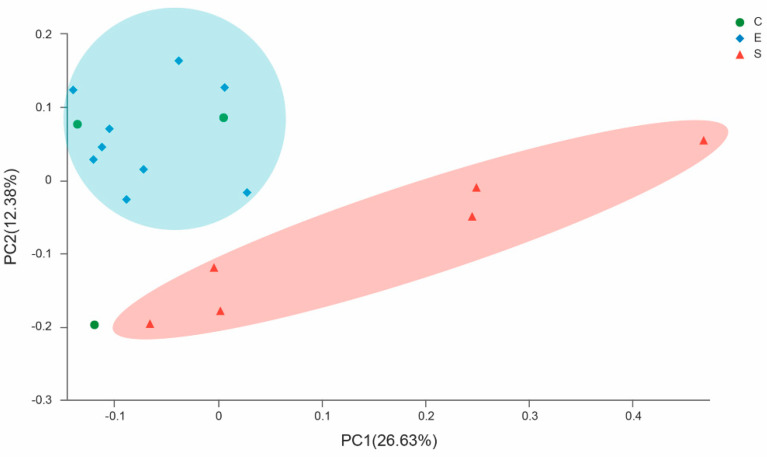
Differences in ruminal bacterial structures among different treatment groups. Principal coordinate analysis (PCoA) was based on the operational taxonomic unit (OTU) data using unweighted and weighted UniFrac distances. Individual samples from C11, C12 and C13 in Group C (green); S11, S12, S13, S21, S22 and S23 in Group S (red); and E11, E12, E13, E21, E22, E23, E31, E32 and E33 in Group E (blue).

Beta diversity analysis was performed in this study. ANOSIM analysis was used to determine if the grouping was meaningful (Table 1). ANOSIM between the different groups receiving dietary supplementation levels of sun-dried mulberry leaves showed no significant differences in rumen microbial community structures at the phylum (*R* = −0.074, *p* = 0.714), genus (*R* = −0.259, *p* = 1.000) or OTU levels (*R* = −0.185, *p* = 1.000). Additionally, no significant differences were observed between rumen microbial community structures at the phylum (*R* = 0.111, *p* = 0.265), genus (*R* = 0.243, *p* = 0.122) or OTU levels (*R* = 0.350, *p* = 0.055) in the cashmere goats in the different groups receiving different supplementation levels of ensiled mulberry leaves. By contrast, the differences in the bacterial communities between different feed types were statistically significant at the phylum (*R* = 0.328, *p* = 0.009), genus (*R* = 0.292, *p* = 0.015) and OTU levels (*R* = 0.336, *p* = 0.01). PCoA analysis based on the unweighted UniFrac distances indicated that the communities of rumen bacteria in Group S were clearly different from those in Group E (Figure 2). Therefore, it was meaningful to divide the experimental treatments into three groups according to different feed types, rather than according to different supplemental levels of mulberry leaves.

### 3.3. Effects of Different Feed Types on the Composition of Ruminal Bacteria

**Figure 3:**
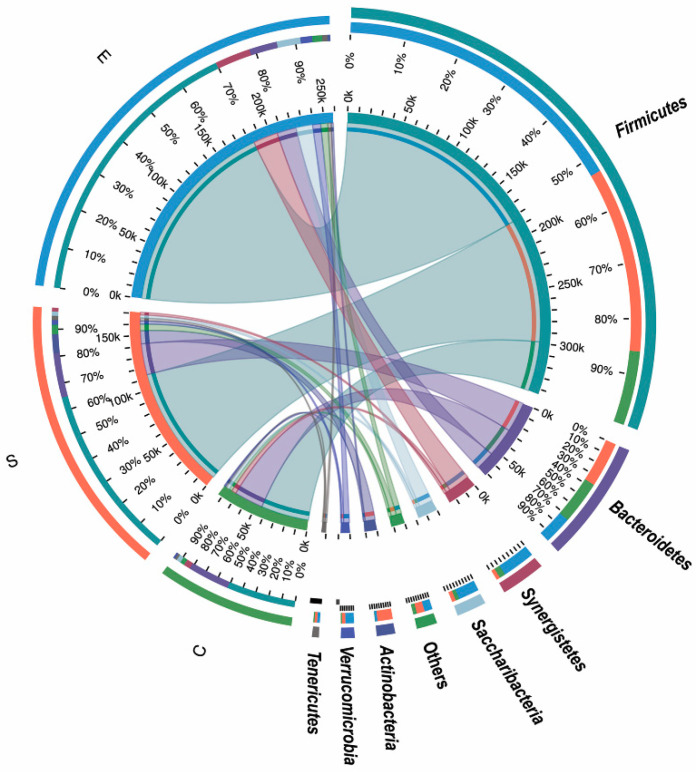
Distribution of the microbial communities in each group at the phylum level. Samples in different groups are on the left side, and the phyla are on the right side. The first and second colorful circles (circles from the outside to the inside): the left half of the circle represents the relative abundance of phyla in different groups, different colors represent different phyla, and the length represents the ratio of the abundance of a phylum in a given group (percentage shown in the second circle); the right half of the circle indicates the proportion of groups in a given phylum, different colors represent different groups, and the length represents the proportion of groups associated with a given phylum (percentage shown in the second circle). The third circle: one end of the color band in the circle is connected to the group from a given phylum (left half circle), the width of the end point of the band indicates the abundance of a different phylum in a given sample, and the other end is connected to the phylum (right half circle). The width of the end point of the band indicates the proportion of different groups in the corresponding phylum.

Of the 19 phyla detected in this study, the four most dominant bacterial phyla were *Firmicutes*, *Bacteroidetes*, *Synergistetes* and *Saccharibacteria* (Figure 3). The most common phyla within Groups C and E were *Firmicutes* (abundances of 52.96% and 71.02%, respectively), *Bacteroidetes* (32.15% and 6.97%), *Synergistetes* (4.98% and 8.75%), *Saccharibacteria* (3.35% and 5.80%), *Lentisphaerae* (1.19% and 2.96%) and *Tenericutes* (1.66% and 1.24%), while the predominant phyla in Group S were *Firmicutes*, *Bacteroidetes*, *Actinobacteria*, *Lentisphaerae*, *Tenericutes* and *Saccharibacteria*, representing 66.22%, 16.84%, 7.08%, 1.69%, 1.63% and 1.58% of the total reads, respectively. There were no significant differences in the proportion of rumen bacterial phyla among three groups, although the proportion of *Actinobacteria* in Group S was increased relative to those in the other groups (>9-fold) (Supplemental Table S5).

**Figure 4:**
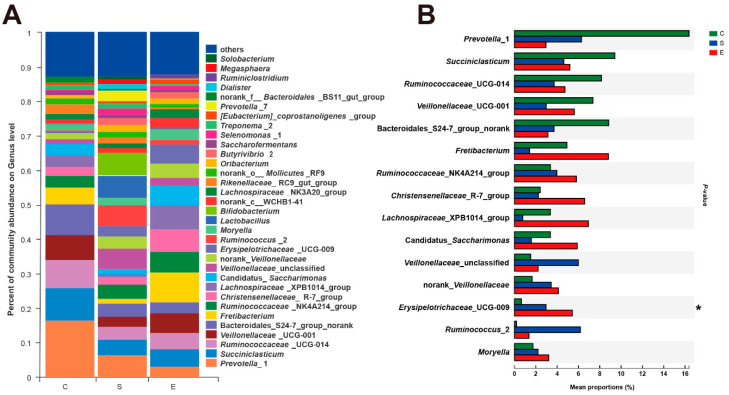
Distribution of genera in different groups. (**A**)The color-coded bar plot represents the average distributions of bacterial genera across the different feed types sampled. Only the dominant genera (those with a relative abundance ≥1%) among rumen bacteria are shown. (**B**) Extended error bar plots illustrate the mean proportions and differences in the bacterial genera in rumen samples. Corrected *p* < 0.05 was marked with *.

A total of 209 taxa (at the genus level) were found in all samples. Among them, the most abundant genera (those with a relative abundance ≥ 1%) are shown in Figure 4A. Within Group C, the dominant bacterial taxa included *Prevotella*_1 (16.30%), *Succiniclasticum* (9.38%), *Bacteroidales*_S24-7_group_norank (8.83%), *Ruminococcaceae*_UCG-014 (8.16%), *Veillonellaceae*_UCG-001 (7.33%), *Fretibacterium* (4.94%) and *Ruminococcaceae*_NK4A214_group (3.36%). In Group S, *Bifidobacterium* (6.38%), *Prevotella*_1 (6.35%), *Lactobacillus* (6.30%), *Ruminococcus*_2 (6.15%), *Succiniclasticum* (4.64%) and *Ruminococcaceae*_NK4A214_group (4.00%) were the most abundant genera. Within Group E, the dominant taxa were associated with *Fretibacterium* (8.74%), *Christensenellaceae*_R-7_group (7.37%), *Lachnospiraceae*_XPB1014_group (6.98%), *Ruminococcaceae*_NK4A214_group (5.80%), Candidatus*_Saccharimonas* (5.80%), *Veillonellaceae*_UCG-001 (5.65%) and *Erysipelotrichaceae*_UCG-009 (5.43%) (Supplemental Table S6). Among these genera, *Prevotella*_1 and *Bacteroidales*_S24-7_group_norank are members of the phylum *Bacteroidetes*, *Fretibacterium* belongs to the phylum *Synergistetes*, and *Bifidobacterium* is a member of the phylum *Actinobacteria*. The other genera belonged to the phylum *Firmicutes* (except for Candidatus*_Saccharimonas*). Notably, the read abundances of *Ruminococcus*_2 in Group S were increased relative to those in the other groups ( >4-fold), and only the relative abundances of *Erysipelotrichaceae*_UCG-009 were significantly increased when mulberry leaves were added to the diets (corrected *p* = 0.045) (Figure 4B). To achieve visualization and clarity, a heatmap depicting the top 100 bacterial genera is shown in Supplemental Figure S1. Meanwhile, the tree along the X-axis in the upper part of Supplemental Figure S1 shows that the community composition in Groups C and E, but not Group S, clustered together.

### 3.4. Functional Predictions of Rumen Bacteria

**Figure 5:**
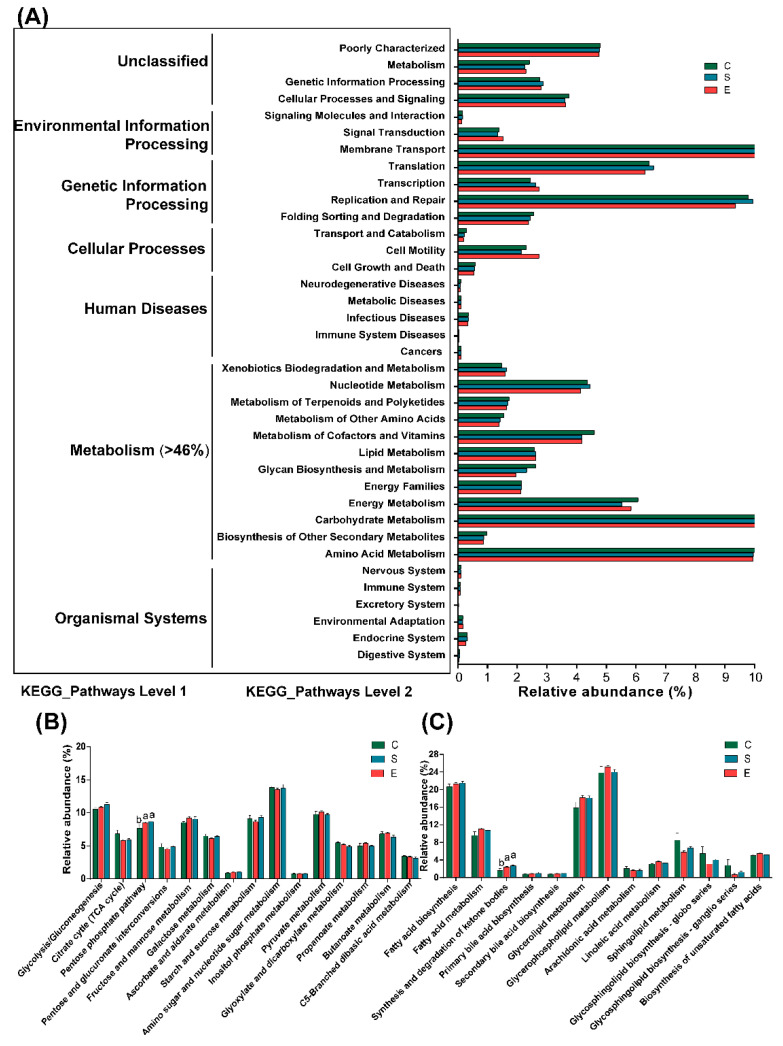
Differences of metabolism function profiles of bacterial community in goats fed different feed types of mulberry leaves used 16S data with PICRUSt. Relative abundance of the predicted different functional taxa at level 1 and 2 (**A**); relative abundance of carbohydrate metabolism (**B**) and lipid metabolism (**C**). The different lowercase letters in the bar chart indicate significant differences in the abundance of different pathways (the corrected *p* < 0.05).

Metabolic functions were predicted using PICRUSt to further understand rumen bacteria (Figure 5A). At KEGG level 1, the highest relative category abundance was “metabolism”, accounting for >46% of all sequence reads (Supplemental Table S7). At KEGG level 2, the majority of genes belonged to membrane transport, amino acid metabolism, carbohydrate metabolism and replication and repair (Supplemental Table S8). In addition, carbohydrate metabolism and lipid metabolism are shown in Figure 5B,C, respectively. Notably, the abundances of sequences involved in the pentose phosphate pathway (corrected *p* = 0.048) (Figure 5B) and in the synthesis and degradation of ketone bodies (corrected *p* = 0.032) (Figure 5C) were significantly decreased in Group C compared to other groups.

### 3.5. Proportion of Selected Microbiota in the Rumen

The qPCR results revealed fluctuations in ciliate protozoa, methanogens and anaerobic fungi among the different rumen samples (Table 2). No significant differences across diets were observed in the numbers of ciliate protozoa, methanogens and anaerobic fungi, respectively (*p* > 0.05).

### 3.6. Rumen Morphological and Functional Parameters

Mulberry leaves had no or only minor effects on the rumen morphological and functional parameters of the rumen in white cashmere goats (*p* > 0.05) (Table 3). The rumen pH was 6.00~6.39, and the proportion of butyrate in Group C had a decreased trend compared with those in the other groups fed mulberry leaves (*p* > 0.05).

### 3.7. Biochemical Analysis of Serum Samples

The effects of mulberry leaves on serum samples are shown in Table 4. Our results revealed that the supplementation of mulberry leaves in the diet could decrease leptin levels (*p* < 0.05), especially between Group C (7.26 ng/mL) and Group S (4.75 ng/mL). There were no significant differences in other serum parameters (*p* > 0.05).

### 3.8. Correlation Analysis

**Figure 6:**
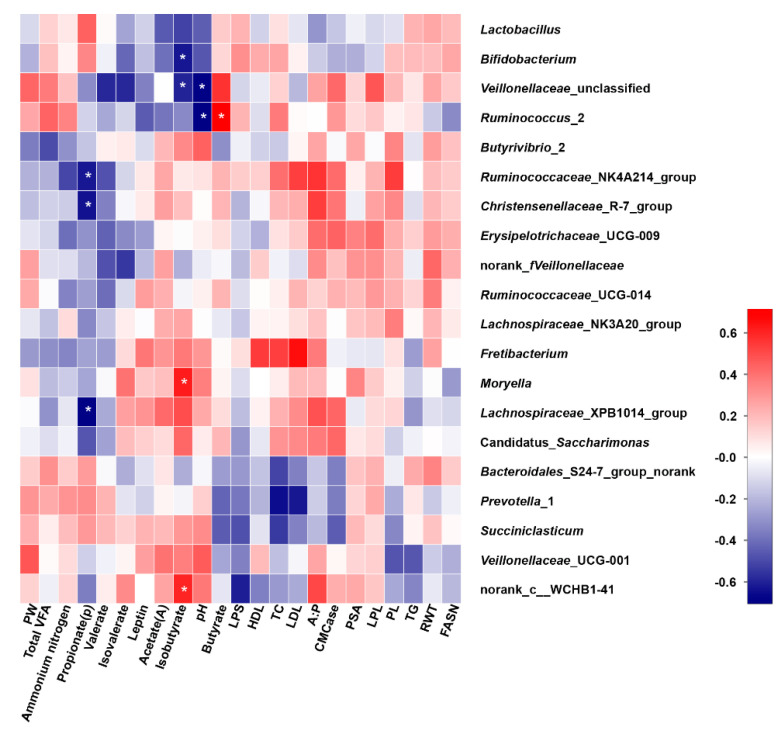
Correlations among rumen morphological parameters, rumen fermentation parameters, CMCase activity, serum biochemical indices and bacterial genera affected by different feed types of mulberry leaves. PL-papilla length, PW-papilla width, PSA-papilla surface area, RWT-rumen wall thickness, CMCase activity-carboxymethylcellulase activity, LPS-Lipase, HDL-high-density lipoprotein cholesterol, LDL-low-density lipoprotein cholesterol, TC-Total cholesterol, LPL-lipoprotein lipase, TG-triglycerides, FASN-fatty acid synthase. Corrected *p* < 0.05 was marked with *.

The Spearman rank correlations were determined among rumen bacteria at the genus level, related fermentation parameters and serum biochemical indices if |r| > 0.55 (Figure 6 and Supplemental Table S9). The relative abundances of the genus *Ruminococcaceae*_NK4A214_group (r = −0.624, *p* < 0.01, corrected *p* < 0.05), *Christensenellaceae*_R-7_group (r = −0.637, *p* < 0.01, corrected *p* < 0.05) and *Lachnospiraceae*_XPB1014_group (r = −0.702, *p* < 0.01, corrected *p* < 0.05) were negatively correlated with the propionate concentration. The isobutyrate concentration was negatively correlated with the relative abundances of the genera *Bifidobacterium* (r = −0.629, *p* < 0.01, corrected *p* < 0.05) and *Veillonellaceae*_unclassified (r = −0.595, *p* < 0.01, corrected *p* < 0.05) but positively correlated with the relative abundance of *Moryella* (r = 0.624, *p* < 0.01, corrected *p* < 0.05). The populations of *Ruminococcus*_2 (r = 0.715, *p* < 0.01, corrected *p* < 0.05) were positively correlated with the butyrate concentration. pH was negatively correlated with the relative abundances of *Veillonellaceae*_unclassified (r = −0.703, *p* < 0.01, *p* < 0.05) and *Ruminococcus*_2 (r = −0.706, *p* < 0.01, corrected *p* < 0.05). In addition, the relationships among the fluctuations in ciliate protozoa, methanogens and anaerobic fungi and fermentation parameters are shown in Supplemental Table S10. The proportion of methanogens was positively correlated with the ratio of acetate to propionate (r = 0.610, *p* < 0.01), whereas the abundance of ciliate protozoa was negatively correlated with acetate (r = −0.641, *p* < 0.01) and butyrate (r = −0.631, *p* < 0.01).

**Figure 7:**
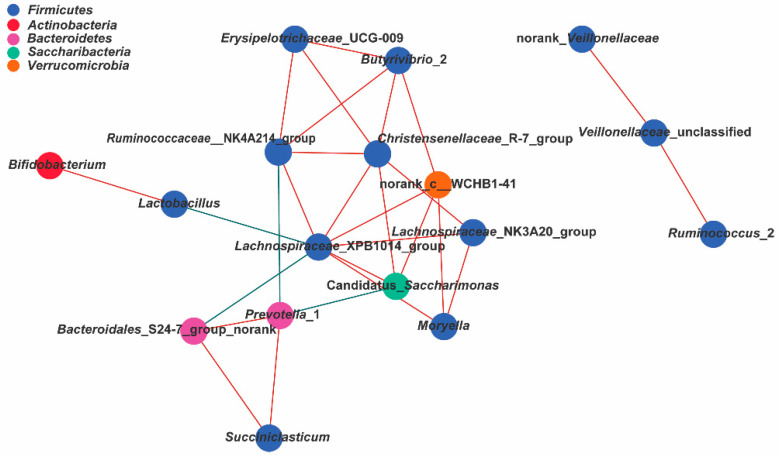
Correlation analysis of bacterial genera was visualized by Cytoscape version 3.6.1, wherein nodes represent bacterial genera, and edges represent significant interactions among nodes (the absolute Spearman coefficients were above 0.55, *p* < 0.05). The node color corresponds to the phylum taxonomic classification. The edge color represents positive (red) and negative (green) correlations.

Our results showed a high level of connectivity within the top 17 bacterial genera (|*r*| > 0.55, *p* < 0.05), which were dominated by phylogenetic similarity (Figure 7). The genus network led to the identification of 17 nodes and 28 edges. Exclusions between *Prevotella_*1 and *Ruminococcaceae*_NK4A214_group (r = −0.564, *p* < 0.05, corrected *p* > 0.05), between *Bacteroidales*_S24-7_group_norank and *Lachnospiraceae*_XPB1014_group (r = −0.605, *p* < 0.05, corrected *p* > 0.05), between Candidatus_*Saccharimonas* and *Prevotella*_1 (r = −0.600, *p* < 0.05, corrected *p* > 0.05), and between *Lactobacillus* and *Lachnospiraceae*_XPB1014_group (r = −0.546, *p* < 0.05, corrected *p* > 0.05) were found in the rumen microbial network. By contrast, significant positive correlations were observed between the other genera shown in Figure 7 (r > 0.55, *p* < 0.05). Notably, corrected *p*-values of the positive associations between *Lactobacillus* and *Bifidobacterium*, between the *Lachnospiraceae*_XPB1014_group and *Ruminococcaceae*_NK4A214_group, between the *Christensenellaceae*_R-7_group *Lachnospiraceae*_XPB1014_group, and between the *Christensenellaceae*_R-7_group and *Ruminococcaceae*_NK4A214_group were less than 0.05. Additionally, the *Lachnospiraceae* _XPB1014_group was the central mode in the network.

## 4. Discussion

### 4.1. Changes in Rumen Fermentation Parameters and Serum Biochemical Indicators

The rumen fluid pH is an important index of rumen health and its normal range is approximately 6~7 [45,46]. Therefore, the rumen fluid pH in this study was within the proper range. The effect of rumen pH on the production of VFA is mainly reflected by the effect on rumen microbial activity [47]. Rumen bacteria convert dietary fiber into VFAs, which provide energy and improve health [48]. As expected, the concentration of butyrate had an increasing trend when a typical TMR was supplemented with mulberry leaves. This was likely related to the increased number of butyrate producing *Erysipelotrichaceae*_UCG-009 [49] in this study after adding mulberry leaves to the diets. Additionally, many studies have shown that leptin concentrations in serum are positively correlated with the proportion of body fat [50,51] and that mulberry leaves can reduce fat deposition [52]. This might be the reason that the leptin concentrations in the present study were decreased when mulberry leaves were added to the diets.

### 4.2. Comparison of the Composition of Ruminal Microbiota after Adding Mulberry Leaves to the Diets

Complex rumen microbial communities play important roles in absorption in the host [40], and their compositions are influenced by diets [53]. The present study revealed the presence of a strong core microbiome among three feed type groups (approximately 69% of total OTUs). Shade and Handelsman [54] reported that such a “core microbiome” was crucial to maintain the “functional redundancy” of the ruminal ecosystem. This redundancy was defined by the ability of a core microbiome to maintain its major functional properties regardless of phylogenetic fluctuations.

Our results revealed the comprehensive structure of rumen microbiota in white cashmere goats fed mulberry leaves. *Firmicutes* and *Bacteroidetes*, which were involved in the degradation of carbohydrates and protein [55], were found to be the two most abundant phyla in this study. Similar findings have been revealed in previous studies [56]. *Proteobacteria* was detected as the third most abundant phylum in previous studies [57], while *Synergistetes* (in Group C and E) and *Actinobacteria* (in Group S) were the third most abundant phyla in this study. The differences in rumen microbial community structures might be related to the animal breed and feed efficiency. In the current study, the abundance of *Actinobacteria*, which belonged to cellulolytic bacteria [58], was increased after adding mulberry leaves to the diet (especially in Group S). Tan et al. [59] also reported that mulberry leaves could increase the population of ruminal cellulolytic bacteria in beef cattle. These results implied that the increasing crude fiber levels after the supplementation of mulberry leaves might increase the number of cellulolytic microbes, which was consistent with our previous finding [37].

The present study also detected the effects of mulberry leaves on the bacterial population at the genus level. These genera changed in response to dietary type due to the specific substrate preferences of these bacteria [60]. Among the identified genera, the populations of *Ruminococcus*_2 in Group S were increased compared with those in the other groups (>4-fold). *Ruminococcus*_2 is a cellulolytic genus that can degrade fiber [61], and, as mentioned previously, higher contents of crude fibers were found in Group S than in the other groups. Furthermore, many studies have reported that mulberry leaves could counteract obesity [52,62], and the abundance of *Erysipelotrichaceae* family was lower in the obesity groups [63]. Hence, the supplementation of mulberry leaves in this study increased the relative abundances of *Erysipelotrichaceae*_UCG-009. In the current study, no effects of mulberry leaves were observed on the number of ciliate protozoa, methanogens and anaerobic fungi, which was similar to the previous studies [64,65]. These observations might suggest that the microbial community was still stable after the goats fed with mulberry leaves.

### 4.3. Relationships among Rumen Microbiota, Fermentation Parameters and Serum Biochemical Indices

The network determined in the current study provided valuable and complementary information for classical enterotype clustering analysis [66]. For both *Lactobacillus* and *Bifidobacterium*, a significantly positive correlation was found by network analysis (r = 0.649, corrected *p* < 0.05), which might be explained by the fact that these two genera could produce lactic acid [61,67]. In accordance with the findings of Zhang et al. [68] regarding the gut microbiota, co-exclusion between the *Lachnospiraceae*_XPB1014_group and *Lactobacillus* was found in the gut microbiota of the present study. Jordan et al. [69] reported that the *Lachnospiraceae* group was a central group in several different locations in the digestive tract, which was consistent with our results. For both *Lachnospiraceae*_XPB1014_group and *Ruminococcaceae*_NK4A214_group, significant positive correlations were found in the network, which could be explained by the fact that these bacterial genera are involved in cellulosic degradation [70]. Additionally, the *Christensenellaceae*_R-7_group was positively correlated with these two groups of fibrolytic bacteria (*Lachnospiraceae*_XPB1014_group and *Ruminococcaceae*_NK4A214_group) in this study, which might imply that *Christensenellaceae*_R-7_group could also help to degrade cellulose. Notably, a large number of ruminal bacteria in goats belonged to unclassified and non-ranking genera. This result showed that Shanbei cashmere goats fed different types of mulberry leaves might have a more abundant ruminal microbe, but only a small portion of the bacteria have been identified with next-generation sequencing technology.

The utilization of nutrients in feed depends mainly on rumen microbiota [53], while the differences in rumen fermentation parameters and serum biochemical indices may provide an indication of feeds [71]. Therefore, one of the purposes of this study was to explore the relationships among rumen microbiota, rumen fermentation parameters and serum biochemical indices. The abundances of the genera *Bifidobacterium* and *Veillonellaceae*_unclassified were negatively correlated with the isobutyrate concentration, while the relative abundance of *Moryella* was positively correlated with the isobutyrate concentration, suggesting that these genera might participate in isobutyrate metabolism. Similarly, in this study, the populations of *Ruminococcus*_2 might also be involved in butyrate metabolism, which was the preferred energy source and exerted a multitude of cellular regulatory effects [72].

### 4.4. Predicted Functions of Ruminal Bacteria in Goats

In addition to the detected changes in microbial abundance, the mulberry leaves might also contribute to maintaining host health by regulating the metabolic pathways of the bacterial microbiota [73]. Based on the PICRUSt prediction for the rumen bacteria, at KEGG level 2, the most abundant gene categories were associated with the functions of membrane transport, carbohydrate metabolism, amino acid metabolism, replication and repair, translation, and energy metabolism, which was similar to the findings in previous studies conducted in goats [74,75]. The high levels of these inferred microbial functions emphasized the vital metabolic functions (i.e., carbohydrate metabolism) for microbial survival [76]. Furthermore, Wang et al. [77] have reported that carbohydrate metabolism and lipid metabolism played important roles in the subclass of category “metabolism” at KEGG level 1. Hence, the effects of mulberry leaves on carbohydrate metabolism and lipid metabolism were presented in this study. The pentose phosphate pathway is a vital pathway for degradation of cellulose [78] and protection from oxidative stress [79]. Dietary crude fiber levels in the present research were increased when mulberry leaves were added to the diets; in addition, mulberry leaves have antioxidant properties [15]. Hence, the pentose phosphate pathway was enhanced when the diets were supplemented with mulberry leaves. The genes associated with the synthesis and degradation of ketone bodies were also increased when mulberry leaves were added to the diets, which might be related to the hypolipidemic capacities of mulberry leaves [52]. This result was consistent with the finding of Kaakoush et al. [80]. Importantly, however, it must be noted that the PICRUSt predictive approach cannot precisely investigate the gene functional contents of bacteria due to the limited number of sequencing studies in ruminants [81]. Thus, further study is needed to provide greater power to examine the effects of mulberry leaves on the functions of bacteria in the rumen of goats. Additionally, although sixty goats (including 10 goats in each treatment) were used in this study, only three goats in each treatment were randomly selected for sampling. In order to improve the accuracy of results, the number of goats sampled should be increased in further studies.

## 5. Conclusions

The effects of mulberry leaves on ruminal microbial communities, ruminal fermentation functions and serum biochemical indices were comprehensively analyzed in white cashmere goats. We observed that although mulberry leaves could cause changes in ruminal microbiota and fermentation parameters, most of the abovementioned indicators did not change significantly, suggesting that the rumen fermentation status was still stable after adding mulberry leaves to the diets. In addition, mulberry leaves only decreased the serum leptin concentrations in goats, which indicated that the supplementation of mulberry leaves could maintain, and even help to promote, goats’ health. Overall, adding mulberry leaves to the diets could keep rumen healthy and result in a potential positive impact on host health, which implied that the feasibility of mulberry leaves as a new feed resource in goats.

## Figures and Tables

**Table 1:** Analysis of similarities (ANOSIM) for rumen microbial composition at the phylum, genus and operational taxonomic unit (out) level.

Groups		*R*			*p*-Value		
Supplemental levels	S (S1,S2)	Phylum	Genus	OTU	Phylum	Genus	OTU
		−0.074	−0.259	−0.185	0.714	1.000	1.000
	E (E1,E2,E3)	Phylum	Genus	OTU	Phylum	Genus	OTU
		0.111	0.243	0.350	0.265	0.122	0.055
Feed types	T *^a^*(C, S, E)	Phylum	Genus	OTU	Phylum	Genus	OTU
		0.328	0.292	0.336	0.009	0.015	0.01

*^a^* T means treatment groups of different feed types.

**Table 2:** Anaerobic fungi, methanogens and ciliate protozoa in the rumen of goats fed different feed types of mulberry leaves.

Taxa *^a^*	Treatment			SEM	*p*
	C	S	E		
Anaerobic fungi	6.30	4.20	5.43	0.330	0.058
Methanogens	4.30	5.31	5.52	0.187	0.066
Ciliate protozoa	7.17	7.17	6.83	0.151	0.568

*^a^* The number of microbes was shown by log_10_ copies/10 ng DNA.

**Table 3:** Effect of mulberry leaf feed types on rumen morphology, fermentation and CMCase activity.

Item			Treatment			SEM	*p*
			C	S	E		
Rumen morphology	PL *^a^*(μm)		747.81	973.86	729.26	61.312	0.186
	PW *^b^*(μm)		301.89	266.44	245.01	10.088	0.127
	PSA *^c^*(μm^2^)		0.13	0.22	0.11	0.197	0.423
	RWT *^d^*(μm)		1545.73	1444.46	1426.49	74.591	0.865
Rumen fermentation parameters	pH		6.37	6.00	6.39	0.086	0.097
	VFA	Acetate(A) (%)	64.27	57.13	60.69	1.046	0.053
		Propionate (P) (%)	18.17	21.13	19.01	0.853	0.435
		Butyrate (%)	12.36	14.90	14.85	0.827	0.557
		Isobutyrate (%)	1.32	1.10	1.46	0.109	0.345
		Valerate (%)	1.42	2.38	1.43	0.296	0.336
		Isovalerate (%)	2.46	2.26	2.55	0.254	0.884
		A:P	3.54	2.70	3.19	0.164	0.375
		Total VFA (mmol/L)	112.67	123.60	95.59	7.004	0.199
	Ammonium nitrogen(mg/dL)		29.38	33.65	27.23	2.468	0.536
CMCase activity *^e^*(U/mL)			3.86	3.46	4.10	0.472	0.847

*^a^* PL-papillae length, *^b^* PW-papillae width, *^c^* PSA-papillae surface area, *^d^* RWT-rumen wall thickness, *^e^* CMCase activtity-carboxymethylcellulase activity.

**Table 4:** The effects of mulberry leaves on serum biochemical indicators.

Items *^a^*	Treatment			SEM	*p*
	C	S	E		
TC (mmol/L)	2.27	2.34	2.57	0.098	0.439
TG (mmol/L)	0.25	0.37	0.25	0.026	0.109
HDL-C (mmol/L)	1.45	1.31	1.39	0.047	0.583
LDL-C (mmol/L)	0.62	0.51	0.60	0.030	0.356
LPS (U/L)	53.17	68.50	59.05	3.710	0.347
LPL (μmol/mL h)	2.19	2.11	2.07	0.119	0.948
Leptin (ng/mL)	7.26	4.75	6.81	0.342	0.003
FASN (ng/mL)	7.97	7.30	7.74	0.331	0.780

*^a^* TC, Total cholesterol; TG, triglycerides; HDL-C, high-density lipoprotein cholesterol; LDL-C, low-density lipoprotein cholesterol; LPS, Lipase; LPL, lipoprotein lipase; FASN, fatty acid synthase.

## Supplementary Materials

The following are available online at https://www.mdpi.com/2076-2607/8/7/981/s1; Figure S1: Heatmap showing the relative abundances of dominant genera; Table S1: Composition and nutrient contents of the experimental diets (%); Table S2: Real time-PCR primers used in this study; Table S3: Diversity estimation based on the 16S rRNA gene libraries for each sample from the sequencing analysis; Table S4: Number of OTUs and diversity estimates based on the 16S rRNA gene libraries from the sequencing analysis; Table S5: The 10 most abundant phyla in the rumen bacteria of goats; Table S6: Distribution of genera in different groups; Table S7: Predicted functions at level 1 of the rumen bacterial microbiota; Table S8: Predicted functions at level 2 of the rumen bacterial microbiota; Table S9: The relationships among bacterial genera, environmental parameters and serum biochemical indices; Table S10: Correlations among the rumen morphological parameters, rumen fermentation parameters, CMCase activity and selected microbiota affected by different feed types.
