# Supplementary material for: Comparison of Rumen Microbiota and Serum Biochemical Indices in White Cashmere Goats Fed Ensiled or Sun-Dried Mulberry Leaves
Source: Microorganisms. 2020 Jun 30;8(7):981. doi: 10.3390/microorganisms8070981 (PMC7409109; doi:10.3390/microorganisms8070981)
Supplement: Supplementary file 1 [file microorganisms-08-00981-s001.zip › Supplementary files/Supplemental Figure S1.docx]

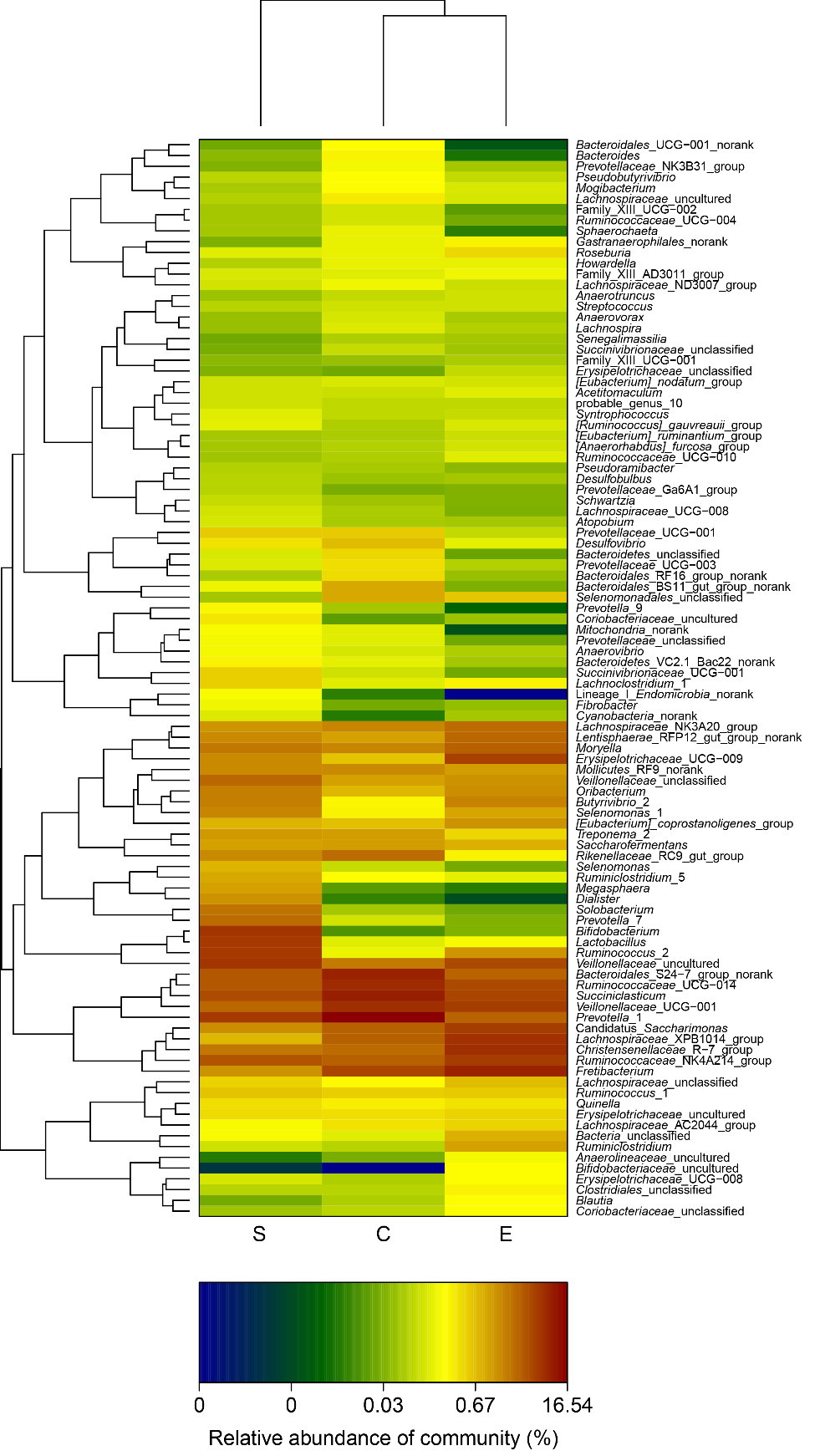


**Figure S1.** Heatmap showing the relative abundances of dominant genera. Hierarchical cluster analysis based on the Bray-Curtis distance of the three groups along the X-axis is shown in the upper part of the figure.
