# Supplementary material for: Comparison of Rumen Microbiota and Serum Biochemical Indices in White Cashmere Goats Fed Ensiled or Sun-Dried Mulberry Leaves
Source: Microorganisms. 2020 Jun 30;8(7):981. doi: 10.3390/microorganisms8070981 (PMC7409109; doi:10.3390/microorganisms8070981)
Supplement: Supplementary file 1 [file microorganisms-08-00981-s001.zip › Supplementary files/Supplemetal Tables/Supplemental Tables S1-S8 and S10.docx]

**Table S1.** Composition and nutrient contents of the experimental diets (%).

| Group *^a^* | C | S | | E | | |
| --- | --- | --- | --- | --- | --- | --- |
|  |  | S1  (10%) | S2  (15%) | E1  (10%) | E2  (15%) | E3  (20%) |
| Ingredients |  |  |  |  |  |  |
| Corn | 27.80 | 22.00 | 19.10 | 28.40 | 28.20 | 31.10 |
| Wheat bran | 7.20 | 7.00 | 6.80 | 6.80 | 7.20 | 7.30 |
| Soybean meal | 4.00 | 3.00 | 2.50 | 3.80 | 3.60 | 2.80 |
| Stone powder | 0.40 | 0.40 | 0.40 | 0.40 | 0.40 | 0.40 |
| CaHPO_4_ | 0.40 | 0.40 | 0.40 | 0.40 | 0.40 | 0.40 |
| 5% Premix *^b^* | 0.15 | 0.15 | 0.15 | 0.15 | 0.15 | 0.15 |
| Silage corn | 15.00 | 5.00 | 0.00 | 5.00 | 0.00 | 0.00 |
| EML *^c^* | 0.00 | 0.00 | 0.00 | 10.00 | 15.00 | 20.00 |
| SML *^d^* | 0.00 | 10.00 | 15.00 | 0.00 | 0.00 | 0.00 |
| Corn stalk | 25.00 | 32.00 | 35.60 | 25.00 | 25.00 | 17.80 |
| Alfalfa powder | 19.80 | 19.80 | 19.80 | 19.80 | 19.80 | 19.80 |
| Salt | 0.25 | 0.25 | 0.25 | 0.25 | 0.25 | 0.25 |
| Total | 100.00 | 100.00 | 100.00 | 100.00 | 100.00 | 100.00 |
| Nutrient level *^e^* |  |  |  |  |  |  |
| DE (MJ • kg ^-1^) | 9.20 | 9.22 | 9.23 | 9.22 | 9.21 | 9.21 |
| CP | 10.52 | 10.52 | 10.52 | 10.52 | 10.52 | 10.52 |
| CF | 17.36 | 19.32 | 20.31 | 17.45 | 17.53 | 17.70 |

*^a^* The Group C samples included goats which were fed with a typical total mixed ration (TMR); the Group S samples included goats which were fed with a typical TMR supplemented with 10% and 15% sun-dried mulberry leaves (Groups S1 and S2); the Group E samples included goats which were fed with a typical TMR supplemented with ensiled mulberry leaves (Groups E1，E2 and E3). These conditions also applied to the analyses described below.

*^b^* Premix contained the following per kg of premix: VA 7 500 IU, VD3 1 050 IU, VE 10 IU, Fe 5 500 mg, Cu 500 mg, Mn 5 000 mg, Zn 4 000 mg, Se 32.5 mg, I 100mg, Co 32.5 mg.

*^c^* EML, Ensiled mulberry leaves.

*^d^* SML, Sun-dried mulberry leaves.

*^e^* The contents of DE and CF were measured values, and the contents of CP was calculated values

**Table S2.** Real time-PCR primers used in this study.

| Target | Primer sequence (5’-3’) *^a^* | Product size (bp) | Annealing temperature  (°C) | References |
| --- | --- | --- | --- | --- |
| Methanogens | F  GGATTAGATACCCSGGTAGT  R GTTGARTCCAATTAAACCGCA | 173 | 60 | [1] |
| Ciliate protozoa | F GCTTTCGWTGGTAGTGTATT  R ACTTGCCCTCYAATCGTWCT | 223 | 55 | [2] |
| Anaerobic fungi | F GAGGAAGTAAAAGTCGTAACAAGGTTTC  R CAAATTCACAAAGGGTAGGATGATT | 120 | 60 | [3] |

*^a^* W = A or T; S = G or C, R = A or G, Y = C or T;

1. Hook, S.E.; Northwood, K.S.; Wright, A.D.; McBride, B.W. Long-term monensin supplementation does not significantly affect the quantity or diversity of methanogens in the rumen of the lactating dairy cow. *Appl. Environ. Microbiol.* **2009,** *75*, 374-380.

2. Sylvester, J.T.; Karnati, S.K.; Yu, Z.; Morrison, M.; Firkins, J.L. Development of an assay to quantify rumen ciliate protozoal biomass in cows using real-time PCR. *J. Nutr.* **2004,** *134,* 3378-3384.

3. Denman, S.E.; McSweeney, C.S. Development of a real-time PCR assay for monitoring anaerobic fungal and cellulolytic bacterial populations within the rumen. *FEMS Microbiol. Ecol.* **2006,** *58*, 572-582.

**Table S3.** Diversity estimation based on the 16S rRNA gene libraries for each sample from the sequencing analysis *^a^.*

| Sample  ID *^b^* | Reads | 0.97 | | | | |
| --- | --- | --- | --- | --- | --- | --- |
|  |  | Ace value | Chao value | Good’s  coverage | Shannon  indices | Simpson indices |
| C11 | 29,123 | 614 | 577 | 0.996223 | 4.15 | 0.0391 |
|  |  | (579,664) | (548,623) |  | (4.13,4.17) | (0.0383,0.0399) |
| C12 | 29,123 | 728 | 711 | 0.996154 | 4.92 | 0.0168 |
|  |  | (699,771) | (682,758) |  | (4.9,4.93) | (0.0164,0.0172) |
| C13 | 29,123 | 740 | 739 | 0.995571 | 4.57 | 0.0295 |
|  |  | (708,785) | (701,800) |  | (4.55,4.59) | (0.0287,0.0304) |
| S11 | 29,123 | 692 | 676 | 0.995742 | 4.28 | 0.0371 |
|  |  | (661,735) | (644,726) |  | (4.26,4.31) | (0.0361,0.038) |
| S12 | 29,123 | 502 | 492 | 0.996944 | 4 | 0.0376 |
|  |  | (477,540) | (466,536) |  | (3.98,4.02) | (0.0369,0.0383) |
| S13 | 29,123 | 528 | 514 | 0.996257 | 3.91 | 0.0371 |
|  |  | (494,578) | (478,573) |  | (3.9,3.93) | (0.0365,0.0377) |
| S21 | 29,123 | 689 | 693 | 0.996189 | 4.8 | 0.0184 |
|  |  | (662,727) | (660,748) |  | (4.78,4.82) | (0.018,0.0189) |
| S22 | 29,123 | 830 | 844 | 0.995296 | 4.91 | 0.0195 |
|  |  | (800,872) | (802,908) |  | (4.89,4.93) | (0.019,0.0201) |
| S23 | 29,123 | 266 | 273 | 0.997940 | 2.14 | 0.2111 |
|  |  | (243,303) | (243,332) |  | (2.12,2.16) | (0.2082,0.2141) |
| E11 | 29,123 | 699 | 721 | 0.994987 | 3.82 | 0.0767 |
|  |  | (656,758) | (661,812) |  | (3.8,3.85) | (0.0748,0.0785) |
| E12 | 29,123 | 703 | 720 | 0.995639 | 4.32 | 0.0398 |
|  |  | (672,746) | (676,790) |  | (4.3,4.34) | (0.0387,0.0408) |
| E13 | 29,123 | 604 | 598 | 0.995938 | 4.27 | 0.03 |
|  |  | (572,650) | (562,657) |  | (4.25,4.28) | (0.0293,0.0306) |
| E21 | 29,123 | 701 | 726 | 0.996086 | 4.77 | 0.0182 |
|  |  | (674,740) | (684,794) |  | (4.76,4.79) | (0.0178,0.0186) |
| E22 | 29,123 | 711 | 716 | 0.995708 | 4.56 | 0.0256 |
|  |  | (680,755) | (676,779) |  | (4.54,4.58) | (0.025,0.0262) |
| E23 | 29,123 | 676 | 671 | 0.995983 | 4.55 | 0.0239 |
|  |  | (648,717) | (638,723) |  | (4.54,4.57) | (0.0233,0.0244) |
| E31 | 29,123 | 564 | 573 | 0.996395 | 3.91 | 0.0575 |
|  |  | (534,608) | (533,638) |  | (3.88,3.93) | (0.0561,0.059) |
| E32 | 29,123 | 703 | 718 | 0.995811 | 4.45 | 0.0303 |
|  |  | (675,743) | (679,780) |  | (4.43,4.47) | (0.0296,0.031) |
| E33 | 29,123 | 717 | 692 | 0.995193 | 4.26 | 0.0327 |
|  |  | (682,766) | (658,745) |  | (4.24,4.28) | (0.032,0.0334) |

*^a^* The operational taxonomic units (OTUs) were defined with 3% similarity.

*^b^* The Group C samples included C11, C12 and C13. The Group E samples included E1 (E11, E12 and E13), E2 (E21, E22 and E23) and E3 (E31, E32 and E33). The Group S samples included S1 (S11, S12 and S13) and S2 (S21, S22 and S23). These conditions also applied to the analyses described below.

**Table S4.** Number of OTUs and diversity estimates based on the 16S rRNA gene libraries from the sequencing analysis.

| Item | Treatment | | | | | | Corrected *p* |
| --- | --- | --- | --- | --- | --- | --- | --- |
|  | C | S | | E | | |  |
|  |  | S1 | S2 | E1 | E2 | E3 |  |
| Good’s coverage | 0.996±0.000 | 0.996±0.000 | 0.996±0.001 | 0.996±0.000 | 0.996±0.000 | 0.996±0.000 | 0.378 |
| ACE indices | 694±40.150 | 574±59.475 | 595±169.461 | 669±32.354 | 696±10.408 | 661±48.834 | 0.426 |
| Chao indices | 676±49.991 | 561±58.015 | 603±170.882 | 680±40.834 | 704±16.915 | 661±44.636 | 0.426 |
| Shannon indices | 4.547±0.223 | 4.063±0.111 | 3.950±0.906 | 4.137±0.159 | 4.627±0.072 | 4.207±0.158 | 0.533 |
| Simpson indices | 0.028±0.006 | 0.037±0.000 | 0.083±0.064 | 0.049±0.014 | 0.023±0.002 | 0.040±0.009 | 0.533 |

**Table S5.** The 10 most abundant phyla in the rumen bacteria of goats.

| Phylum (%) | Treatment | | | SEM | Corrected *p* |
| --- | --- | --- | --- | --- | --- |
|  | C | S | E |  |  |
| *Firmicutes* | 52.96 | 66.22 | 71.02 | 3.028 | 0.630 |
| *Bacteroidetes* | 32.15 | 16.84 | 6.97 | 3.772 | 0.510 |
| *Synergistetes* | 4.98 | 1.44 | 8.75 | 1.551 | 0.510 |
| *Saccharibacteria* | 3.35 | 1.58 | 5.80 | 0.795 | 0.510 |
| *Lentisphaerae* | 1.19 | 1.69 | 2.96 | 0.508 | 0.772 |
| *Tenericutes* | 1.66 | 1.63 | 1.24 | 0.377 | 0.887 |
| *Spirochaetae* | 1.44 | 1.32 | 0.48 | 0.287 | 0.630 |
| *Proteobacteria* | 1.55 | 1.32 | 0.44 | 0.221 | 0.510 |
| *Actinobacteria* | 0.26 | 7.08 | 0.75 | 1.931 | 0.510 |
| Bacteria_unclassified | 0.15 | 0.23 | 0.91 | 0.263 | 0.630 |

**Table S6.** Distribution of genera in different groups.

| Genus (%) | Treatment | | | SEM | Corrected *p* |
| --- | --- | --- | --- | --- | --- |
|  | C | S | E |  |  |
| *Prevotella*_1 | 16.30 | 6.35 | 3.01 | 2.443 | 0.446 |
| *Succiniclasticum* | 9.38 | 4.64 | 5.16 | 1.123 | 0.511 |
| *Fretibacterium* | 4.94 | 1.42 | 8.74 | 1.553 | 0.446 |
| *Veillonellaceae*_UCG-001 | 7.33 | 2.97 | 5.65 | 0.856 | 0.446 |
| *Ruminococcaceae_*UCG-014 | 8.16 | 3.72 | 4.72 | 1.053 | 0.511 |
| *Christensenellaceae*_R-7_group | 2.72 | 2.26 | 7.37 | 0.952 | 0.446 |
| *Ruminococcaceae*_NK4A214_group | 3.36 | 4.00 | 5.80 | 0.703 | 0.511 |
| *Lachnospiraceae*_XPB1014_group | 3.35 | 0.80 | 6.98 | 1.324 | 0.446 |
| *Bacteroidales*_S24-7_group_norank | 8.83 | 3.68 | 3.12 | 1.451 | 0.511 |
| Candidatus _ *Saccharimonas* | 3.35 | 1.57 | 5.80 | 0.795 | 0.446 |
| *Erysipelotrichaceae*_UCG-009 | 0.66 | 1.63 | 5.43 | 0.934 | 0.045 |
| *Ruminococcus*_2 | 0.18 | 6.15 | 1.34 | 1.690 | 0.511 |
| *Moryella* | 1.75 | 2.19 | 3.22 | 0.490 | 0.585 |
| *Veillonellaceae*_unclassified | 1.13 | 2.86 | 1.45 | 0.440 | 0.446 |
| norank*_ Veillonellaceae* | 1.66 | 3.44 | 4.13 | 0.322 | 0.686 |
| *Bifidobacterium* | 0.01 | 6.38 | 0.03 | 1.936 | 0.511 |
| *Lactobacillus* | 0.15 | 6.30 | 0.22 | 1.452 | 0.446 |

**Table S7.** Predicted functions at level 1 of the rumen bacterial microbiota.

| Functions (%) | Treatment | | | SEM | Corrected *p* |
| --- | --- | --- | --- | --- | --- |
|  | C | S | E |  |  |
| Metabolism | 48.40 | 47.22 | 46.60 | 0.309 | 0.167 |
| Genetic Information Processing | 21.17 | 21.54 | 20.74 | 0.135 | 0.100 |
| Environmental Information Processing | 11.94 | 13.25 | 14.22 | 0.332 | 0.100 |
| Cellular Processes | 3.17 | 2.91 | 3.46 | 0.141 | 0.264 |
| Human Diseases | 0.71 | 0.69 | 0.64 | 0.012 | 0.155 |
| Organismal Systems | 0.73 | 0.74 | 0.68 | 0.019 | 0.277 |

**Table S8.** Predicted functions at level 2 of the rumen bacterial microbiota.

| Functions (%) | Treatment | | | SEM | Corrected *p* |
| --- | --- | --- | --- | --- | --- |
|  | C | S | E |  |  |
| Membrane transport | 10.43 | 11.78 | 12.60 | 0.313 | 0.166 |
| Amino acid metabolism | 10.37 | 9.96 | 9.94 | 0.082 | 0.166 |
| Carbohydrate metabolism | 10.05 | 10.47 | 10.41 | 0.129 | 0.394 |
| Replication and repair | 9.78 | 9.94 | 9.36 | 0.097 | 0.110 |
| Translation | 6.44 | 6.59 | 6.30 | 0.056 | 0.166 |
| Energy metabolism | 6.06 | 5.53 | 5.83 | 0.081 | 0.166 |
| Poorly characterized | 4.80 | 4.77 | 4.76 | 0.024 | 0.595 |
| Metabolism of cofactors and vitamins | 4.60 | 4.19 | 4.17 | 0.077 | 0.166 |
| Nucleotide metabolism | 4.36 | 4.45 | 4.14 | 0.051 | 0.110 |
| Cellular processes and signaling | 3.75 | 3.61 | 3.64 | 0.031 | 0.323 |

**Table S10.** Correlations among the rumen morphological parameters, rumen fermentation parameters, CMCase activity and selected microbiota affected by different feed types *^a^.*

| Items | | | Methanogens | Anaerobic fungi | Ciliate protozoa |
| --- | --- | --- | --- | --- | --- |
| Rumen morphology | PL | | 0.335 | -0.280 | -0.162 |
|  | PW | | -0.179 | 0.104 | -0.377 |
|  | PS | | -0.022 | -0.160 | -0.214 |
|  | PT | | -0.244 | -0.067 | -0.168 |
| Rumen fermentation  parameters | pH | | -0.244 | 0.253 | 0.380 |
|  | VFA | Acetate (A) | 0.492 | -0.024 | -0.641** |
|  |  | Propionate(P) | -0.032 | 0.079 | 0.127 |
|  |  | Butyrate | 0.422 | -0.119 | -0.631** |
|  |  | Isobutyrate | -0.026 | 0.470* | 0.245 |
|  |  | Valerate | -0.368 | 0.036 | 0.589* |
|  |  | Isovalerate | -0.123 | 0.437 | 0.294 |
|  |  | Total VFA | 0.064 | 0.098 | -0.180 |
|  |  | A:P | 0.610** | 0.135 | -0.362 |
|  | Ammonium nitrogen | | -0.032 | 0.079 | 0.127 |
| CMCase activity | | | 0.492 | -0.024 | -0.641** |

*^a^ p* < 0.05 is marked with *, *p* < 0.01 is marked with **.
